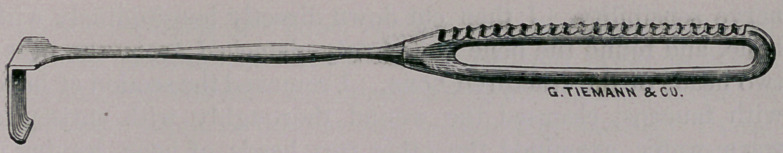# Extroversion of Ihe Bladder

**Published:** 1896-04

**Authors:** A. E. Spohn

**Affiliations:** Corpus Christi, Tex.


For the Texas Medical Journal.

### EXTROVERSION OF THE BLADDER.

BY A. E. SPOHN, M. D., CORPUS CHRISTI, TEX.

I DO NOT know of any deformity which can entail upon its possessor more real suffering, mentally and physically, than an extrophy of the bladder. This condition, fortunately, is rare. Yet common enough to be considered one of the recognized deformities.

I first saw the subject of this report, fourteen years ago, just after birth; and when a few months of age, made an unsuccessful attempt to close the opening. I advised his parents to wait until the child was old enough to appreciate his condition, when he could co-operate in my efforts to relieve him. I did not see him again until April, 1894, a fine looking young man, well developed, of more than ordinary intelligence, anxious to assist me in any way, even suffer, to get relief.

During the intervening period of twelve years; he had been a constant sufferer, dressed like a girl; for it would have been impossible to clothe him otherwise. His bladder protruded, a fungoid looking mass, the size of a large orange, bleeding when touched, and extremely sensitive. The ureters opened on either side of this mass, keeping the parts, as well clothing, constantly bathed with urine; he was offensive, and really an object of pity; shunned by his friends and associates, what condition could have been worse! Young as he was, for a long time, he had been trying to make up his mind to destroy himself and end his misery.

He was exceedingly fine looking, indeed handsome, and I sometimes fancied he was rather feminine in build, though he was decidedly masculine. His mammary glands were well developed, he had broad hips, and his gait was that of a female. I can account for the broad hips and peculiar gait, by an absence of the pubic arch, and his resemblance to a female may have been caused by his having been only partially developed, his penis being only $\frac{3}{4}$ of an inch in length, and the testicles very small, rudimentary.

He had no navel, the abdomen being open from where the navel should have been, the opening extending to the end of the penis, with bladder protruding, i. e., extroverted. I could trace, or rather locate the openings of the ureters on either side of the protruding mass, by drying it with cotton, could also see the openings at the neck of the bladder, if it could be called a neck, for the penis was very short and broad, with the prepuce, also open, hanging like a collar below it. His testicles had only partially descended, and it was not until I had commenced forcing the bladder in, that I could tell whether he had testicles or not, for which reason there was some doubt as to his sex, though I never questioned it. I could invert the bladder, but as soon as the pressure used, a ball of cotton, was removed, it would come out again. I hoped to be able to restore the bladder to its normal position, restore the abdominal wall, unite the dorsum of the penis, and at least, be able to leave him in such condition that he could use an instrument to receive his urine.

Before attempting to close the abdominal opening, I instructed my nurses to keep the bladder inverted, and held in that position, using round pads of absorbent cotton, replacing them when saturated with urine. These pads were as large as could be conveniently inserted, and in a few weeks I succeeded in very much enlarging the bladder, also reducing the fungoid condition; in fact, I had the bladder in the form of a little pocket, which I kept carefully filled with borated cotton, using a solution of cocaine to the part when very painful. I next drew the abdominal opening together with adhesive plaster, in which I was quite successful, and in a couple of months I had the part in condition to attempt the closure of the opening, with at least some chance for success. I split the skin around the opening, making two flaps; one composed of skin and mucous membrane, of what I thought belonged to the bladder, the other skin and underlying tissues, dissecting this flap well from the under one, so as to slide the sides, or outer flaps together, thus giving better support. The under flaps I turned down, bringing the edges carefully together, using fine catgut sutures, buried, put in after the method of Lembert. I then brought the outer flaps together, using deep silkworm gut sutures, so introduced as to bring the muscular tissues together, supporting all with strips of adhesive plaster. At this first operation I closed the opening as far as the base of the bladder,

FIG. 1.

1. Extroverted bladder. 2. Penis. 3. Prepuce. 4. Line of incision just outside of mucous membrane. 5. Inner flap turned in and united with Lembert sutures of fine cat buried. 6. Outer flap dissected up and united with silk-worm gut sutures, supported by adhesive strips. 7. Openings of ureters. 8. Incision along floor of penis to enlarge urethra. 9. Incision on either side of urethra. 10. Upper part of scrotum.

leaving the lower opening for drainage. This operation was only partially successful, bridging the opening by three firm bands, the balance failing to unite; however sufficient to support the bladder and keep it back, still requiring support by adhesive strips, put on so as to double the edges of opening in and relieve the bands from tension. I continued the splitting operation until I had all the openings closed, but one, corresponding to the center of the bladder, which I left open purposely for drainage, when ready to close the dorsum of the penis. The next operation was done at the neck of the bladder. I could see the openings of the ducts, and split as deeply as possible at this point, hoping to include in my stitches some muscular fibres, and closed the part as before, stitching the lower flap with fine catgut Lembert sutures, the outer flap with silkworm gut. This operation bridged across the membranous portion or rather base of the bladder; for the part from the bladder to end of the penis was not more than one inch and a half in length. After this operation I gave my patient a rest of two months, as his courage was failing, and it was only after great persuasion I could get him to continue the treatment, as he had already taken chloroform ten or twelve times. I next closed the penis. This organ was very small, and it seemed quite impossible to have the urethra of sufficient size; consequently I found it necessary to make an incision from the meatus along the floor of the urethra to the base of the bladder, and by splitting the sides of the penis at junction of urethral mucous membrane with the skin, united the edges of the mucous membrane, using buried Lembert sutures of fine catgut, over a small glass tube extending the whole length of the urethra; the skin was brought together with fine silk. While closing the penis the bladder was drained through the opening left for the purpose. The other openings were also closed by splitting operations, having chosen this method to avoid any loss of tissue. He now passes his urine through the urethra, which is large enough to admit a No. 8 catheter, but is compelled to use a rubber instrument during the day; at night he empties his bladder every two or three hours. He could do so during the day, but with the desire to urinate, the urine comes away. I consider the result in this case an exceedingly good one, and think, in time he may have control of his bladder. It required an enormous amount of labor on my part part, and suffering on his; still in his case the end has more than justified the means. The little fellow was under the influence of chloroform fifteen times, and received many a nick without it; not taking into consideration the various operations tried with cocaine, the treatment lasting over two years. I did not follow, or consult any method in this case, but simply tried to approximate tissues in as natural a position as possible, draining the urine from parts recently united, until the operation was complete.

Fig. 2.

Result showing part completely covered; what appears as a navel is where outer flaps come together. Penis is very small urethra admits a No. 8 catheter.

This cut represents Dr. Spohn's "new principle in surgical instruments," described in our last issue. The seriated back enables the surgeon always to know just where the cutting edge is, when operating in a cavity. It applies to all instruments. Tremain, the manufacturer, thinks well of it.

## Figures and Tables

**Fig. 1. f1:**
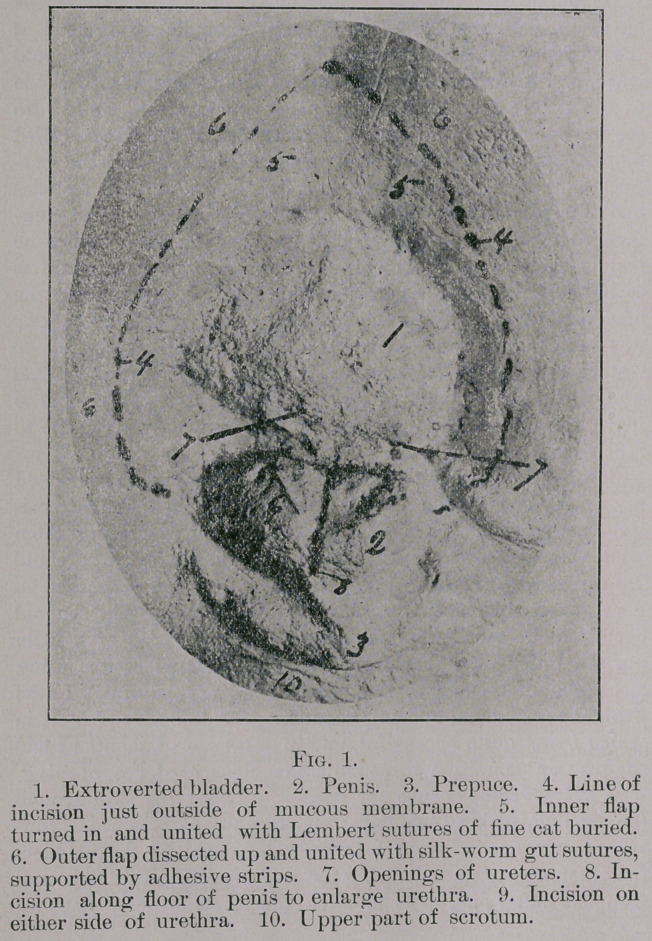


**Fig. 2. f2:**
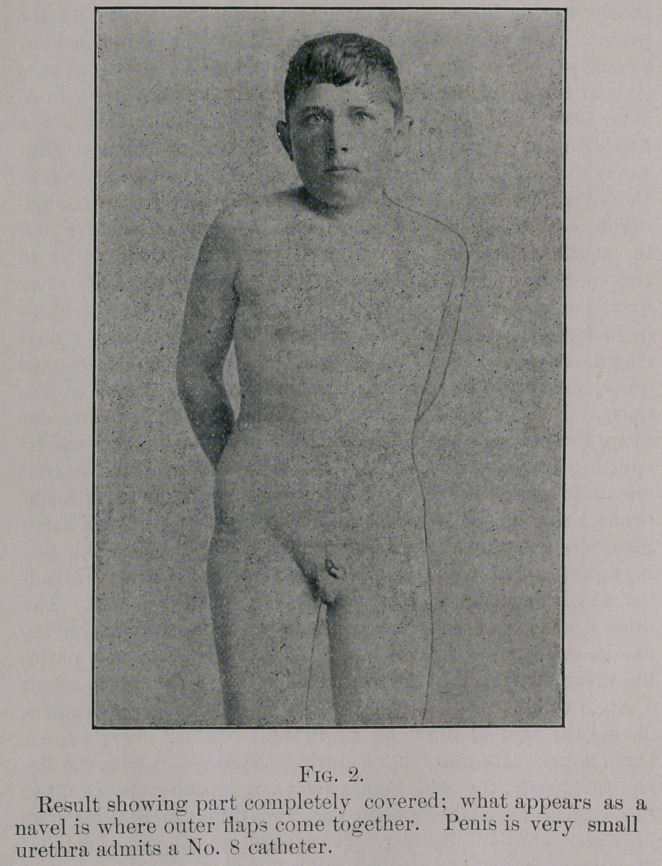


**Figure f3:**